# The role of prostate tumor overexpressed 1 in cancer progression

**DOI:** 10.18632/oncotarget.14104

**Published:** 2016-12-22

**Authors:** Verónica Cánovas, Matilde Lleonart, Juan Morote, Rosanna Paciucci

**Affiliations:** ^1^ Biomedical Research Group of Urology, Vall d’Hebron Research Institute, Pg. Vall d’Hebron 119-129, 08035 Barcelona, Spain; ^2^ Biomedical Research in Cancer Stem Cells, Vall d’Hebron Research Institute, Pg. Vall d’Hebron 119-129, 08035 Barcelona, Spain; ^3^ Deparment of Urology, Vall d’Hebron Hospital, Pg. Vall d’Hebron 119-129, 08035 Barcelona, Spain; ^4^ Universitat Autònoma de Barcelona, Spain

**Keywords:** PTOV1, cancer progression, prostate cancer, cancer stem cells, transcription

## SUMMARY

Prostate-Tumor-Overexpressed-1 (PTOV1) is a conserved adaptor protein discovered as overexpressed in prostate cancer. Since its discovery, the number of binding partners and associated cellular functions has increased and helped to identify PTOV1 as regulator of gene expression at transcription and translation levels. Its overexpression is associated with increased tumor grade and proliferation in prostate cancer and other neoplasms, including breast, ovarian, nasopharyngeal, squamous laryngeal, hepatocellular and urothelial carcinomas. An important contribution to higher levels of PTOV1 in prostate tumors is given by the frequent rate of gene amplifications, also found in other tumor types. The recent resolution of the structure by NMR of the PTOV domain in *PTOV2*, also identified as Arc92/ACID1/*MED25*, has helped to shed light on the functions of PTOV1 as a transcription factor. In parallel, by studying its interaction with RACK1, we have discovered PTOV1 action in promoting mRNAs translation. Here, we will focus on the role of PTOV1 in cancer, re-examine its pro-oncogenic effects and re-evaluate the most relevant interactions and evidences of its cellular functions. The data are used to formulate a model for the mechanisms of action of PTOV1 in line with its recently described activities and cellular pathways modulated in cancer.

## THE GENE STRUCTURE

Prostate Tumor Overexpressed-1 (PTOV1) was first described as gene and protein overexpressed in prostate tumors and preneoplastic lesions of high grade intraepithelial neoplasia (HGPIN) [1]. The gene was assigned to chromosome 19q13.3 by FISH analysis on human metaphase chromosomes. This region harbors a large number of androgens modulated and prostate cancer (PC) related genes, including the proteases prostate specific antigen, kallikrein 1 (KLK1), kallikrein related peptidase 2 (KLK2) [2], the apoptotic regulator BCL2 associated X (BAX) [3], kallikrein related peptidase 11 (TLSP) [4] or kallikrein related peptidase 6 (Zyme/Neurosin) [5].

**Figure 1:**
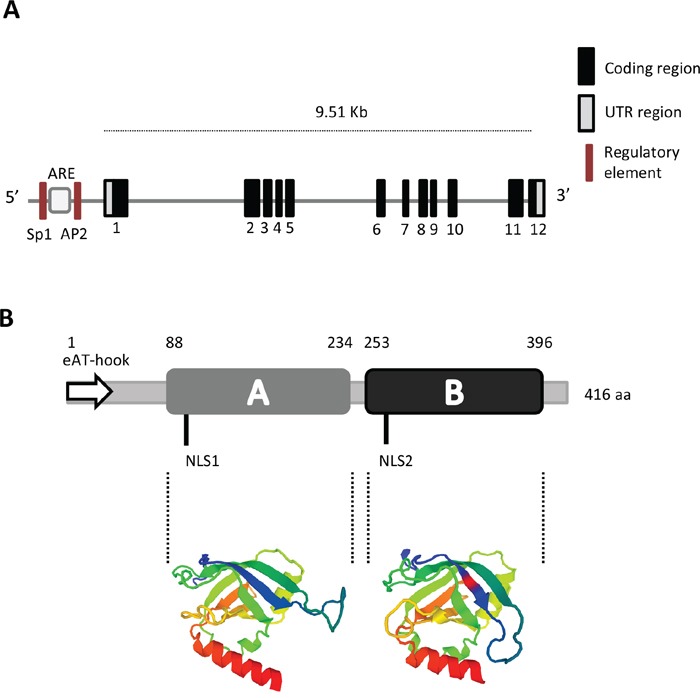
Gene and protein structure of PTOV1 **A**. The gene includes 12 coding exons and two untranslated regions (UTR). In the putative promoter region, the localization of regulatory sites for ARE (Androgen Responsive Element), SP1 (Specificity protein 1), and AP2 (Activator protein 2) are shown (not in scale). **B**. Protein organization showing the A and B domains, the Nuclear Localization Sequences (NLS1 and NLS2) and the extended AT-hook (eAT-hook) motif (not in scale). The three-dimensional structure of the A and B domains based on the Swiss Model, is shown in color [13].

The gene *PTOV1* spans 9.51 Kb and includes 12 exons of which exons 3 to 6 code for the first *PTOV* homology block, or A domain, and exons 7 to 12 for the second block, or B domain [1] (Figure 1A). At least two transcripts resulting from differential splicing have been described: the first one includes 1,875 bps and translates for a protein of 416 residues, the second one includes 1,539 bps and translate for protein of 374 amino acids [6]. Two alternative splicing events at the 5’ and 3’ ends in the latter transcript result in a shorter protein with different N-terminal and C-terminal residues. These differences likely affect the function of the final product.

A related gene containing one *PTOV*-domain, identified as *PTOV2*, was discovered on the same chromosome, 14 Kb upstream from the putative first exon of *PTOV1* [1]. *PTOV2* was later found to correspond to the subunit 25 of the Mediator complex, MED25, composed of up to thirty proteins, that is an essential component of the eukaryotic RNA polymerase II complex regulating eukaryotic transcription [7–9]. The Mediator functions as a transcription coactivator in all eukaryotes. It is also referred as the vitamin D receptor interacting protein (DRIP) coactivator complex and the thyroid hormone receptor-associated proteins (TRAP) [10]. It is required for the transcription of most class II genes in yeasts and mammals and is essential for activator-dependent transcription. The Mediator acts as a bridge between the RNA polymerase II and the activator transcription factors [10]. MED25 was also identified as p78/Arc92/ACID1 [8, 11]. In this review, we will refer to PTOV2 as MED25.

The orthologous *PTOV1* gene in *Drosophila melanogaster* (acc. AC013074) shows a similar modular arrangement, but a lower degree of similarity between the two domains A and B (33% identity; 57% similarity) [1]. The orthologous *MED25* gene, found on chromosome 3R at 3Mb from *PTOV1*, was also predicted to encode a protein with a single PTOV block embedded within an 863-amino acid protein.

The putative promoter region of *PTOV1* reveals the presence of consensus sequences for transcription factors SP1 and AP2 and a putative Androgen Responder Element (ARE), similar to those observed in the *PSA* gene. In agreement with these observations, PTOV1 expression is androgen-responsive [1]. This has been confirmed in vascular smooth muscle cells (VSMCs) where the gene was suggested to play a critical role in androgen related atherogenesis in the human aorta through the regulation of proliferation of neointimal VSMCs [12].

## THE PROTEIN STRUCTURE

PTOV1 is an adaptor, conserved in vertebrates (mammals and fish) and in arthropods (insects), although not in fungi (yeasts). The protein interacts with a number of factors both in the nucleus and the cytoplasm to regulate gene expression at transcription and posttranscriptional levels and to promote cancer cell proliferation and motility.

The predicted protein, 416 aminoacids long, reflects the structure of the gene and presents two highly homologous domains arranged in tandem, identified as A domain (146 amino acids) and B domain (143 amino acids), that show 66% identity and 79% similarity among each other [1, 13] (Figure 1B). The protein presents two putative nuclear localization signals (NLS), one in the A domain and the second in the B domain. Recent studies using NMR technology revealed the structure of the PTOV domain of MED25 [14–16]. This domain (391–543) exhibits high sequence identity with the A and B domains of human PTOV1 (81% and 73%, respectively). The secondary structure of MED25 forms a seven-stranded β-barrel framed by three α-helices of topology A(↑)B(↓)D(↑)G(↓)F(↑)E(↓)C(↑) in which strands C, E, F and G are flanked by α-helices I and III, and helix II connects strand D and E at one end of the barrel [15, 16]. MED25 also presents two different positively electrostatic charged regions that appear to be important for its binding to the trans activation domain (TAD) of the RNA Polymerase II.

The PTOV domain in MED25 shares structural similarity with the β-barrel domains of the Ku70/Ku80 heterodimer and the Spen paralog and ortholog C-terminal (SPOC) domain of SMRT/HDAC1-associated repressor protein (SHARP) [14–16]. These proteins are involved in transcriptional activation, double-stranded DNA break repair, and transcriptional repression by interaction with histone deacetylase complexes. For instance, SHARP has been identified as a component of the Notch co-repressor complex [17]. The PTOV domain shares the central β-barrel and two flanking helices with the SPOC domain in SHARP, but differs from the SPOC domain by the absence of four helices that flank the barrel, the presence of a C-terminal helix α3 with a unique location and a long loop connecting β1 and β2 [16]. These observations suggest that the PTOV domain in MED25 and PTOV1 might have a crucial function in chromatin remodeling and for connecting the activity of several transcription factors to the RNA Polymerase II complex. However, in contrast to MED25, PTOV1 did not interact with the Mediator complex [11].

Very recently, an extended (e)AT-hook motif comprising the first N-terminal 43 amino acids was identified in PTOV1, suggesting a function as nucleic acid binding protein (Figure 1B) [18]. Classical AT-hook motifs in proteins can bind nucleic acids at AT-rich sequences in the minor groove of the DNA, and have been described as characteristics of proteins associated with chromatin remodeling, histone modifications, chromatin insulator and proposed to anchor chromatin-modifying proteins [19, 20]. However, the (e)AT-hook discovered at the N-terminal of PTOV1 is a new functional AT-hook-*like* motif that differs in the basic amino acid patches from the canonical G-R-P (glycine-arginine-proline) core. This motif showed higher RNA binding affinity compared to DNA [18], and its deletion resulted in a stronger signal of the mutated protein in the nucleus. These apparently contrasting observations may be explained considering the following findings. We have previously shown that the nuclear localization of a GFP-PTOV1, where the first 56 amino acids comprising the (e)AT-hook were replaced by GFP, did not significantly accumulate in the nucleus, suggesting that other features, such as two conserved NLS signals, might be responsible for the localization of the (e)AT-hook deleted PTOV1 [21, 22]. In addition, we have also shown that PTOV1 enters the nucleus at the beginning of the S- phase of the cell cycle [21]. Furthermore, the higher affinity of the (e)AT-hook for RNA may suggest that PTOV1 can be found in ribonucleic-complexes that shuttle their carriers (mRNA) from the nucleus to the cytoplasm, in agreement with its interaction with RACK1 in ribosomes. Thus, the accumulation of the (e)AT-hook deleted PTOV1 in the nucleus observed by Filarsky and collaborators [18], might be the result of the cell type used, the cycle phase at the time of observations, and/or the lack of ribonucleic complexes formation that would shuttle PTOV1 and its cargo to the cytoplasm.

## PTOV1 EXPRESSION IN NORMAL AND CANCER TISSUES

*PTOV1*, was suggested to be one of the genes most discerning between normal and carcinomatous prostate [23]. Most functions of PTOV1 have been surmised in pathological conditions, such as cancerous cells and tissues. However, its expression has also been detected in normal tissues (human brain, heart, skeletal muscle, kidney, liver, ovary, aorta and salivary gland), although little has been described about its function at those sites [1, 12, 24, 25]. The expression of PTOV1 in human abdominal aorta and VSMCs cells is androgen-responsive and may play a role in androgen related atherogenesis [12]. In normal epithelial cells from different tissues, PTOV1 is mostly undetectable or shows a weak staining [21, 26, 27]. However, few sporadic prostatic luminal cells in isolated glands display intense staining, mostly cytoplasmic and in some cases also nuclear. These PTOV1-positive cells also stain for chromogranin A, suggesting their neuroendocrine origin [21].

In pre-neoplastic lesions of High Grade Prostate Epithelial Neoplasia, HGPIN, associated with prostate carcinomas, PTOV1 expression is increased compared to the normal prostate epithelium [28, 29]. This higher expression in HGPIN was found helpful to discriminate those premalignant lesions associated with cancer, suggesting the potential value of PTOV1 detection in the early diagnosis of PC [28]. Of interest, the proportions of Ki-67 positive nuclei and the levels of PTOV1 in HGPIN areas adjacent to cancer lesions are higher than those found in HGPIN areas away from the cancer, supporting the concept of *field cancerization* or *field effect* in prostatic carcinogenesis [29, 30]. More recently, the expression of PTOV1 in atypical adenomatous hyperplasia (AAH), a proliferative lesion of the transition zone of the prostate that morphologically resembles low grade carcinoma, has been associated with PC [31].

**Figure 2:**
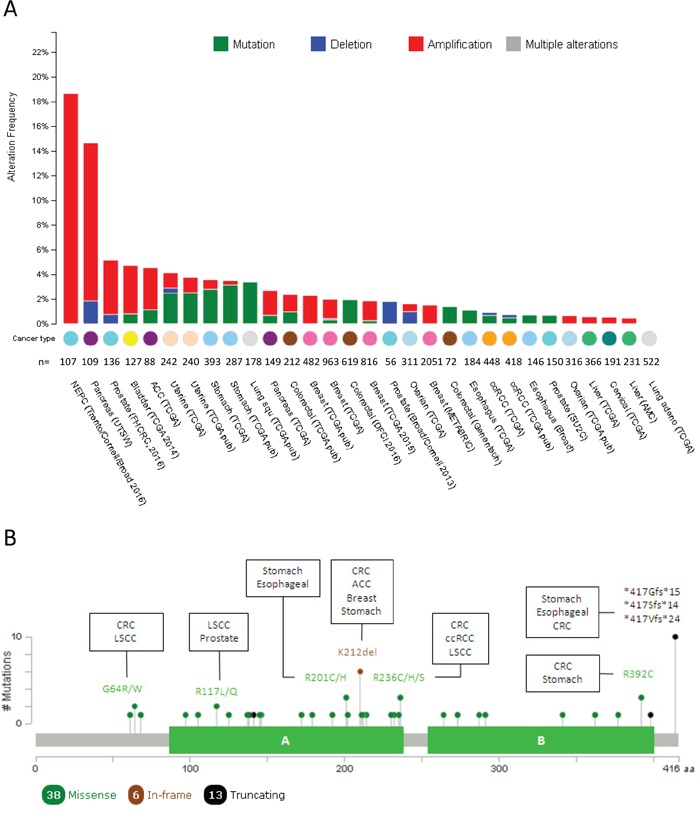
Mutational landscape of PTOV1 across human cancer **A**. Histogram representing the amplifications, deletions and mutations of PTOV1 in different types of tumors according to cBioPortal data. Tumor tissue, sample size and datasets information employed for the analyses are listed below each bar. NEPC: neuroendocrine prostate cancer; ACC: Adrenocortical carcinoma; ccRCC: clear cell renal carcinoma **B**. Schematic picture of the protein organization showing the mutational profile (localization, type and frequency of each mutation) across human tumors. Filled circles represent the position of the mutation and color indicates the type of mutation. The description of the most frequent mutations and those tumors in which such mutation was found are specified. *: nonsense mutation; fs: frameshift mutation; LSCC: lung squamous cell carcinoma.

In prostate adenocarcinoma 71% of T2 and T3 stages overexpressed PTOV1 [21]. This overexpression was limited to the cytoplasm in 59% of samples whereas a strong expression was detected both in the nucleus and the cytoplasm in the remaining samples, with a small proportion of tumors showing a strong nuclear staining and weak cytoplasmic staining. More recently, metastatic primary tumors and metastatic lesions were shown to express significantly higher levels of PTOV1 compared to non-metastatic tumors [32, 33]. This high expression of PTOV1 significantly associated to the Ki67 index suggesting its participation in an active proliferative status. Remarkably, this relationship was stronger in tumors with nuclear PTOV1 staining. These findings are supported by observations *in vitro*, where in quiescent PC cells, PTOV1 localized in the cytoplasm but after serum stimulation it partially translocated to the nucleus at the beginning of the S phase [21]. The transfection of PTOV1 forced PC3 cells to enter the S phase with a subsequently increase in the levels of cyclin D1, indicating that the overexpression of PTOV1 can directly increase the proliferation of PC cells. In addition, PTOV1 is required for the growth and full metastatic potential of PC cells *in vivo* [32, 33]. An important contribution to higher levels of PTOV1 in aggressive prostate tumors might be given by the high rate of PTOV1 amplifications, also found in other tumor types, as shown by analyses of publicly available genomic datasets (Figure 2A) [34, 35]. Significantly, the highest frequency of amplifications (above 18%) is found in metastatic lesions of adenocarcinomas and neuroendocrine prostate tumors (Figure 2A) [36]. Because very high expression of PTOV1 in sporadic luminal cells in prostate glands associated to cancer very likely correspond to neuroendocrine cells [21], altogether the above findings suggest that PTOV1 expression might be connected to the aggressive features of neural subtypes of PC defined by expression profiles [37, 38].

More recently, numerous reports have described the overexpression of PTOV1 in different types of tumors. These included tumors of the breast, pancreas, liver, colon, kidney, bladder, laryngeal, cerebral gliomas and ovary [25–27, 39–43]. The association between its overexpression and high grade of malignancy was strong in primary hepato-cellular carcinoma, epithelial ovarian cancer, breast cancer and clear cell renal carcinomas, where PTOV1 expression was closely correlated with the clinic-pathological characteristics and tumor aggressiveness. In addition, a significant association between high levels of PTOV1 and unfavorable prognosis and poor survival has been observed in several types of carcinomas. In breast cancer, 99.4% of the cancer samples analyzed expressed PTOV1, of which 49.1% showed high expression. The median survival of patients with high PTOV1 levels was 78 months *versus* 115 months in patients with low PTOV1 expression [27]. In laryngeal squamous cell carcinoma, PTOV1 expression correlates with advanced clinical stage and it was shown to be an independent predictor of overall survival and progression-free survival [41]. HPV-positive head and neck cancers have better prognosis than HPV-negative cancers [43–45]. In this context, the levels of expression of PTOV1 in combination with the infection status with the human papillomavirus (HPV) could predict outcome in early-stage laryngeal squamous cell carcinoma [41]. Association to a better outcome was observed in HPV-positive/PTOV1-negative subgroup. In contrast, the HPV-negative/PTOV1-positive subgroup showed the worst outcome.

Similarly to prostate carcinomas, the subcellular distribution of PTOV1 in breast cancer and nasopharyngeal carcinomas was detected both in the nucleus and cytoplasm of carcinomatous cells [21, 27, 39]. In aggressive prostate tumors, PTOV1 stained intensely in the nucleus of local and distal (bones) metastatic cells [33]. In urothelial carcinoma (UC) nuclear staining was significantly more frequent compared to benign tissue and a reduced cytoplasmic expression significantly correlated with higher pathological stage and grade, suggesting a functional shift for PTOV1 from the cytoplasm to the nucleus in the progression of these tumors [42]. These observations are in line with previous reports showing that intense nuclear PTOV1 expression was able to distinguish in a significant manner high-grade urothelial carcinoma [40]. Thus, the nuclear presence of PTOV1 might be critical for proliferation and tumor progression, suggesting that its transcription regulating abilities are relevant functions.

As mentioned above, gene amplifications for PTOV1 are frequent in prostate carcinomas, pancreas and present in other tumors (Figure 2A). In addition, mutations are also frequent in several tumors types. About 57% of the mutations are localized inside the A domain (Figure 2B). The majority are missense mutations, more frequent in colorectal, stomach, lung squamous cell carcinoma, esophageal, and breast cancer. Most of the missense mutations have a predicted functional impact score neutral or low according to Mutation-Assessor [46]. However, the R117L/Q mutation found in LSCC and prostate cancer respectively, has a predicted functional impact score of medium, suggesting it might be a critical change for the function of PTOV1 in these tumors. A recurrent in frame mutation (K212del) is found in adenocortical carcinoma, colorectal, breast and stomach carcinoma.

## PTOV1 TRANSCRIPTIONAL REGULATORY FUNCTIONS

Homology models of MED25 PTOV and human PTOV1 have shown that amino acid residues involved in TAD binding in MED25 are generally conserved in PTOV1, suggesting that both domains may serve as activator-binding modules [16]. However, in contrast to MED25, PTOV1 did not interact with the Mediator complex [11]. The differential biological activities of PTOV in MED25 and PTOV1 were suggested to be due to modulation of protein-protein interactions patterns by some amino acid residues which are differently grouped peripherally around the charged surface region [14, 15]. It was described that the retinoic acid (RA) activates the response of its receptor (RAR) using MED25 that, through its VWA domain, interacts with the Mediator and, through the PTOV domain, binds to the activators (histone acetyltransferase CBP) [47]. These observations suggest a role for the PTOV domain of MED25 in chromatin remodeling and pre-initiation complex assembly to recruit activators to the basal transcriptional machinery [11]. Additional evidences confirmed that the domain PTOV of MED25 is responsible for binding the TAD domain of several transcription factors, including ERM/ETV5, a PEA3 member of ETS-related transcription factors [48], the nuclear receptor Hepatocyte Nuclear Factor 4 alpha [49], the transcription factor ATF6 alpha [50], a master regulator of endoplasmic reticulum (ER) stress response genes, the retinoic acid receptor (RAR) [51], STAT6 and chromatin remodelers [52]. Because PTOV1, in contrast to MED25, did not interact with the Mediator complex [11], its action may plausibly modulate, or hamper, the MED25 PTOV module binding the activator in those cells where it is overexpressed, as described below for the *Retinoic Acid Receptor* (*RAR*). In agreement with the above observations, PTOV1 was recently identified as a regulator of transcription of several genes, including *RAR, HES1, HEY1*, and *Dickkopf-1*.

### The retinoic acid receptor promoter

**Figure 3:**
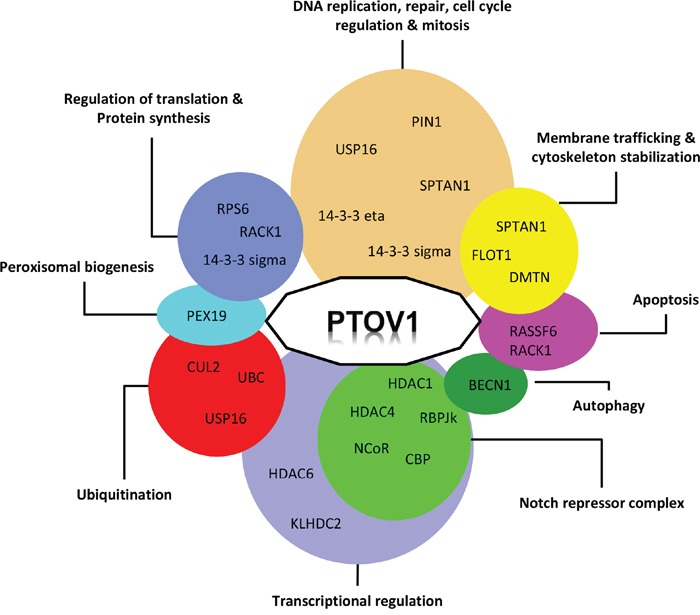
Functional interaction network of PTOV1 Proteins interacting with PTOV1 are clustered according to their proposed cellular functions.

Retinoids are promising chemotherapeutics that inhibit cell growth by inducing apoptosis, senescence and differentiation of cancer cells [53, 54]. Unfortunately, intrinsic or acquired resistance to these agents frequently occurs after cancer therapy [55]. The formation of the complex MED25-RAR and RA, induced the stimulation of *RAR* promoter activity [47]. PTOV1 was shown to suppress the MED25-enhanced *RAR* activity by binding the activator CREB-binding protein (CBP) (Table 1 and Figure 3) [51]. Thus, the expression of PTOV1 prevented CBP binding to MED25 inducing a repression of the *RAR* promoter. CBP belongs to a family of large multifunctional transcriptional coactivators that through their acetyl transferase action modify histones and other proteins, regulating a large number of transcription activators and cellular functions (Table 1) [56]. Both PTOV1 and MED25 proteins interact with the acetyl transferase CBP through the PTOV domain [32, 51]. Chromatin IP (ChIP) assays showed that PTOV1 itself is not recruited to the RA-responsive *RARβ2* promoter. Instead, the increased PTOV1 expression inhibited CBP chromatin binding by forming a chromatin-free PTOV1-CBP interaction that sequesters away CBP from MED25 [51]. In this context, in response to RA the LIM family member Zyxin was shown to interact and cooperate with PTOV1 in RAR repression, by forming a ternary complex with CBP and PTOV1 that antagonized MED25 for CBP binding [57]. These data suggest a potential molecular mechanism for PTOV1 in RA resistance.

**Table 1:** PTOV1 interaction partners

Interactor	Experimental evidence	Dataset	Description
BECN1	Affinity capture-MS	[122]	Beclin 1. Plays a key role in autophagy as a component of the phosphatidylinositol-3-kinase (PI3K) complex which mediates vesicle-trafficking processes in multiple cellular processes, including tumorigenesis, neurodegeneration and apoptosis [123, 124].
CBP/CREBBP	Co-IP	[32, 47]	cAMP-response element binding protein (CREB) binding protein. It is involved in the transcriptional coactivation of many different transcription factors. It plays critical roles in embryonic development, control of growth, and homeostasis by coupling chromatin remodeling to transcription factor recognition. The protein encoded by this gene has intrinsic histone acetyltransferase activity and also acts as a scaffold to stabilize additional protein interactions with the transcription complex. Acetylated histones and non-histones proteins may act as a transcriptional activator [56, 125, 126].
CUL2	Affinity capture-MS	[127]	Cullin2. Core component of multiple cullin-RING-based ECS (ElonginB/C-CUL2/5-SOCS-box protein) E3 ubiquitin-protein ligase complexes, which mediate the ubiquitination of target proteins [128].
DMTN/EBP49	Two hybrid	[129]	Dematin actin binding protein/erythrocyte membrane protein band 4.9. Cytoskeletal protein that bundles actin filaments in a phosphorylation-dependent manner. Dematin deletion has been reported in prostate tumors [130, 131].
FLOT1	Co-IPCo-localizationCo-fractionation	[22]	Flotillin-1. It associates to lipid rafts and localizes to non-caveolar membranes. This protein plays a role in vesicle trafficking, cell morphology and signaling. Elevated expression of FLOT1 has been reported in different types of tumors. Its nuclear localization is associated to induction of proliferation. FLOT1 regulates the function of Aurora B kinase in metaphase cells [111, 113, 116]
HDAC1	Pull-down	[32]	Histone deacetylase 1. It is a component of the histone deacetylase complex which plays a key role in regulation of eukaryotic gene expression. Together with metastasis-associated protein-2 (MDM2), it deacetylates p53 and modulates its effect on cell growth and apoptosis. It is an element of the Notch repressor complex [32, 132].
HDAC4	Pull-down	[32]	Histone deacetylase 4. It possesses histone deacetylase activity and represses transcription when tethered to a promoter. It is an element of the Notch repressor complex. Inhibitors of function increase cytotoxicity of docetaxel in gastric cancer cells [133, 134]
HDAC6	Protein-peptide	[100]	Histone Deacetylase 6. Deacetylates lysine residues on the N-terminal part of the core histones. Histone deacetylation gives a tag for epigenetic repression alters chromosome structure and affects transcription factor access to DNA and plays an important role in transcriptional regulation, cell cycle progression and developmental events. The protein contains an internal duplication of two catalytic domains which appear to function independently of each other [100, 135].
KLHDC2	Affinity capture-MS	[136]	Kelch domain containing 2. Represses CREB3-mediated transcription by interfering with CREB3-DNA binding [137]
NCoR	Pull-down assay	[32]	Nuclear receptor corepressor 1. Transcriptional repressor of thyroid-hormone and retinoic-acid receptors. It is part of a complex which also includes histone deacetylases and other transcriptional regulators [138]
PEX19	Affinity capture-MS	[136]	Peroxisomal biogenesis factor 19. Required for early peroxisomal biogenesis [139]
PIN1	Two hybrid	[129]	Peptidylprolyl cis/trans isomerase, NIMA-interacting 1. It catalyzes the cis/trans isomerization of peptidyl-prolyl peptide bonds. This PPIase specifically binds to phosphorylated ser/thr-pro motifs to catalytically regulate the post-phosphorylation conformation of its substrates. The conformational regulation catalyzed by this PPIase has a profound impact on key proteins involved in the regulation of cell growth, genotoxic and other stress responses, the immune response, induction and maintenance of pluripotency, germ cell development, neuronal differentiation, and survival [140–142].
GNB2L1/RACK1	Two hybridCo-localizationCo-IP	[33]	Guanidine Nucleotide Binding protein (G protein), beta polypeptide 2-like 1. Interacts with PKC isoforms and ribosomes. It is involved in different cellular processes including apoptosis, cell spreading, cell proliferation, regulation of insulin receptor and resistance to drugs [94, 97]
RASSF6	Affinity capture-MS	[143]	Ras association (RalGDS/AF-6) domain family (N-terminal) member 9. Involved in the induction of apoptosis. It may act as a Ras effector protein [144].
RBP-Jκ/CBF-1	Co-IPPull-down assay	[32]	Recombination signal binding protein for immunoglobulin kappa J region. Is a transcriptional regulator in the Notch signaling pathway. It acts as a repressor when not bound to Notch proteins and an activator when bound to Notch proteins. It is thought to function by recruiting chromatin remodeling complexes containing histone deacetylase or histone acetylase proteins to Notch signaling pathway genes [32, 145].
RPS6	Co-IPCo-localizationCo-fractionation	[33]	Ribosomal protein S6. It is a component of the 40S subunit. It is the major substrate of protein kinases in the ribosome. Phosphorylation is induced by a wide range of stimuli, including growth factors, tumor-promoting agents, and mitogens. Dephosphorylation occurs at growth arrest. The protein may contribute to the control of cell growth and proliferation through the selective translation of particular classes of mRNA. Overexpressed in esophageal squamous cell carcinoma, renal cell carcinoma metastases and hyperphosphorilated in lung cancer [146–148].
SFN/14-3-3 sigma	Affinity capture-MS	[149]	Stratifin. Adapter protein implicated in the regulation of numerous signaling pathways including cancer metabolic reprogramming. When bound to KRT17, regulates protein synthesis and epithelial cell growth by stimulating Akt/mTOR pathway. It is a p53-regulated inhibitor of G2/M progression [150, 151].
SPTAN1	Two hybrid	[152]	Spectrin, alpha, non-erythrocytic 1. Filamentous cytoskeletal protein involves in the stabilization of the plasma membrane and organization of intracellular organelles. Also involved in DNA repair, cell cycle regulation, cell migration and its deletion is related to some encephalopaties [152, 153]
UBC	Reconstituted complex	[154]	Ubiquitin C. Polyubiquitin precursor. Provides extra ubiquitin during stress and it loss cannot be compensated by induction of the other Ub genes [155].
USP16	Protein-peptide	[100]	Ubiquitin specific peptidase 16. Deubiquitinates histone H2A. Regulates kinetochore localization of Plk1 to promote proper chromosome alignment in mitosis [156].
YWHAH/14-3-3 eta	Affinity capture-MS	[157]	Tyrosine 3-monooxygenase/tryptophan 5-monooxygenase activation protein, eta. Adapter protein implicated in the regulation of numerous signaling pathways. Role in mitotic progression, potentially a therapeutic target for cancers [158, 159].

The table summarizes the interactors reported for PTOV1 based on STRING [120] and BIOGRID [121]. It describes the experimental evidences for each interaction and includes a brief explanation on the role reported for the interacting partner. IP: immunoprecipitation; MS: mass spectrometry.

### The HES1 and HEY1 promoters

Notch is an evolutionarily conserved signaling pathway that regulates cell fate, tissue homeostasis, cell differentiation, proliferation and growth [58]. Activation of the Notch receptor induces its entrance to the nucleus where it acts as a transcription factor for numerous targets, including *HES1* and *HEY1* genes, two well known downstream regulators of the pathway [59, 60]. In the absence of activated Notch a transcriptional repressor complex, that include SMRT/NCoR and HDAC1, is formed on target promoters [61].

Aberrant Notch signaling has been detected in different types of cancer to suppress or activate cancer progression depending on the cell context and tumor type [62–64]. In PC, its role in progression has been studied *in vitro* and *in vivo* with contradictory results [65–67]. We have shown that PTOV1 in metastatic prostate tumors is significantly overexpressed and represses the transcription of the downstream targets of Notch, the *HES1* and *HEY1* genes, by interacting with SMRT, RBP-Jκ, NCoR, HDAC1 and HDAC4 (Figure 3) [32]. PTOV1 is bound to the chromatin of these promoters when Notch is inactive. Its repressive action was reverted by trichostatin A (TSA), an HDAC inhibitor, indicating the requirement for the activity of HDACs. Interestingly, the repression by PTOV1 was abolished by the overexpression of CBP, in agreement with previous reports showing that *HES1* transcription was activated by CBP [68]. PTOV domains shares structural similarity with the SPOC domain of SHARP, a known component of the Notch repressor complex [17, 69], and thus PTOV1 may be a facultative additional Notch co-repressor restricted to cancerous events. Additional *in vivo* evidence supports the role of PTOV1 as a negative regulator of the Notch pathway [32]. In the *Drosophila melanogaster* wing model, the expression of the human PTOV1 exacerbated Notch deletion mutant phenotypes and suppressed the effects of constitutively active Notch. In human tissues, the normal prostate epithelium revealed high levels of expression of HES1 and HEY1 proteins, supporting activated Notch signaling, whereas metastatic samples expressed significantly lower levels of these proteins, suggesting a Notch repressed state [32]. In contrast, the expression of PTOV1 in the normal prostate epithelium was mostly absent, but the protein was significantly overexpressed in metastatic samples. In human PC cell lines, the downregulation of PTOV1 induced an upregulation of the endogenous *HEY1* and *HES1* genes, and reciprocally, the ectopic expression of PTOV1 in PC cells and HaCaT keratinocytes, where Notch acts as tumor suppressor, caused the inhibition of expression of *HEY1* and *HES1* genes [70, 71]. All together, these observations support a pro-oncogenic role for PTOV1 as a negative regulator of Notch signaling in PC progression. They also support a tumor-suppressor function of Notch in PC, similarly to previous reports in skin, myeloid leukemia, and cervical carcinoma cells [62, 70, 72].

### The dickkopf-1 promoter

Aberrant activation of Wnt/β-catenin signaling was reported in breast cancer and strong evidence point to a possible epigenetic silencing of negative regulators of Wnt, although the regulatory mechanisms underlying these epigenetic changes are poorly understood [73]. Very recently, PTOV1 expression was shown to activate Wnt/β-catenin signaling in breast cancer [74]. In the canonical Wnt pathway, binding of Wnt ligand to frizzled receptors and lipoprotein receptor-related protein-5 or 6 (LRP5/6) co-receptors initiates a cascade, which results in β-catenin activation, its nuclear translocation and transcription of target genes [75]. *Dickkopf-1* (*DKK1*) is negative regulator of Wnt signaling. Silencing its expression was tightly associated with DNA hypermethylation and histone deacetylation [76, 77]. *DKK1* methylation has been reported in 27% of breast cancer cell lines and 19% of breast cancer patients [78].

In human breast carcinoma, high levels of PTOV1 expression correlated with high levels of nuclear β-catenin and low levels of DKK1 [74]. The overexpression of PTOV1 in breast cancer cell lines induced the nuclear translocation of β-catenin and increased β-catenin/TCF transcriptional activity. PTOV1 overexpression repressed *DKK1* transcription via the recruitment of HDACs to the promoter and a concomitant decrease of histone acetylation [74]. Treatment with TSA reverted the repression of *DKK1*. These findings suggest a role for PTOV1 as a novel epigenetic regulator of the Wnt/β-catenin pathway in breast tumorigenesis.

## ROLE OF PTOV1 IN CANCER STEM CELLS

Extensive research over many years have posited a cancer stem cell (CSC) model for tumorigenesis in which a small proportion of cells, possibly originated from normal stem cells, reside inside tumors and may be subject of additional genetic changes or epigenetic transitions that can drive tumor initiation, progression to metastasis and drug resistance [79, 80]. These cells, identified and isolated in a number of solid cancers models, including prostate cancer, have self-renewing and differentiation abilities and are capable of recapitulating the characteristics of the original tumor from which they were isolated [81]. As the normal stem cells, CSCs possess the ability to grow *in vitro* as self-renewing spheres. However, the mechanisms implicated in CSCs self-renewal and differentiation, critical to understand CSCs biology and their role in tumorigenesis, are still under investigation. In the prostate, recent studies have shown that CSCs can also be isolated from cancer cell lines, and the analysis of their transcriptome shows expression profiles that support CSCs identity [82].

Recently, it was shown that PTOV1 promotes the *in vitro* formation of spheres in HaCaT transformed keratinocytes and PC3 prostate cancer cells, and promoted tumor growth *in vivo* [32, 33]. In addition, in breast cancer cells PTOV1 was shown to enhance tumor growth, increase the number of mammospheres, the proportion of the side population (SP), and of CD24^-^/CD44^+^ cells [74]. These findings reveal the ability of the protein PTOV1 to promote CSCs-*like* properties in cells from different tumor types. Significantly, the associated overexpression of the protein in more aggressive tumors with poor prognosis provides support for a role of PTOV1 in favoring CSCs self-renewal and tumor progression.

## ROLE OF PTOV1 IN PC RESISTANCE TO CHEMOTHERAPY

Because of the high resistance of metastasis to conventional androgen-depletion-therapy (ADT) and chemotherapy, metastatic PC is virtually incurable [83]. The numerous evidences that PTOV1 is expressed significantly more in aggressive tumors and metastatic lesions and its implication in the mechanisms leading to cancer progression, suggested a potential action of this protein in recurrence. Indeed, PTOV1 is expressed at higher levels in cells resistant to chemotherapy with docetaxel compared to parental sensitive cells (Cánovas et al., submitted 2016). The ectopic overexpression of PTOV1 in docetaxel sensitive PC cells resulted in a significant increase in the number of prostatospheres and a significantly greater capacity of the cells to survive to docetaxel treatment (Cánovas et al., submitted 2016). These effects are associated to a significant increase in the levels of genes associated with the resistance to docetaxel *(ABCB1, CCNG2, TUBB4A, TUBB2B*) and with the stemness phenotype (*LIN28A*, *ALDH1A1, MYC, NANOG*). In turn, the knockdown of PTOV1 very significantly inhibited self-renewal and proliferation in all cell models. In addition, cells knockdowns for PTOV1 showed a striking cell cycle arrest at the G2/M phase that was associated to a significant increase in apoptosis. Docetaxel resistant cells knockdown for PTOV1 showed more extreme phenotypes. These findings identify PTOV1 as a promoter of docetaxel resistance and a survival factor for CRPC cells and give support to potential anticancer therapies directed to eliminate the action of this protein in future therapeutic interventions.

## SEARCH FOR PTOV1 INTERACTING PROTEINS TO UNVEIL ITS FUNCTIONS

Discovering new proteins interactions can be helpful in defining the functions in which a protein of interest is involved. PTOV1 has been detected at different subcellular locations, including sub-membrane sites, lipid rafts, cytoplasm especially the perinuclear region, and the nucleus. These locations are likely associated with different interacting proteins. A summary of the proteins interacting with PTOV1 is given in Table 1. These proteins are involved in several cellular processes, although it is not clear how each interaction contributes to a role of dysregulated PTOV1 expression in cancer progression. Figure 3 summarizes the interactions of PTOV1 with other proteins in relationship with their described functions. Groups including transcriptional regulation and DNA replication, cell cycle regulation-mitotic functions contain numerous interactors. Similarly, groups including protein synthesis, ubiquitination and membrane trafficking functions contain each three interactors. Together, these observations indicate that PTOV1 participates in different cellular events at different subcellular locations. Interactions described by high-throughput capture affinity assays that lack further functional analyses will not be reviewed here. Those interactions studied more in detail, where some functional aspects are revealed, are reviewed below.

### Receptor of activated protein C kinase, RACK1

We have described the interaction of PTOV1 with RACK1, also known as guanine nucleotide binding protein beta polypeptide 2-like 1, GNB2L1 [33]. RACK1 contains seven repeats of a short Trp-Asp [W-D] dipeptide, highly conserved in eukaryotes, and although was first described to interact with Protein Kinase C (PKC) isoforms [84, 85], it is now known to interact with numerous proteins and is involved in a diverse array of cellular processes. These include apoptosis regulation of insulin receptor and IGF-1R signaling, cell spreading, cell proliferation, STAT3 activation, and UV radiation [86–88]. In 2004, an interesting report described RACK1 as part of the 40S small ribosomal subunit, and localized the protein at the head of the 40S subunit close to the mRNA exit channel [89], suggesting that it may function as a molecular link connecting cell signaling with the protein translation machinery. Later, RACK1 was described to recruit active PKCβII on ribosomes to phosphorylate eukaryotic initiation factor 6 (eIF6) that allows the assembly of the 80S ribosome on the pre-initiation mRNA complex [90, 91]. RACK1 has been described as an adaptor required for the PKC-mediated phosphorylation of Ser129 of JNK [92]. More recently, the recruitment of JNK by RACK1 has been identified as part of a mechanism underlying the quality control of newly synthesized proteins under stress conditions [93]. After stress induction, RACK1 recruits activated JNK to 40S on actively translating ribosomes to phosphorylates the elongation factor eEF1A2, which in turn promotes the ubiquitination and degradation of damaged newly synthesized polypeptides [93].

RACK1 overexpression has been reported in different tumors and it was found to be a differential diagnostic biomarker and predictor for poor clinical outcome in breast and pulmonary carcinomas [94–96]. In hepatocellular carcinoma it promotes chemoresistance and tumor growth by localization on ribosomes and phosphorylation of eukaryotic initiation factor 4E (eIF4E), preferential translation of cyclin D1, Myc, survivin and Bcl-2 [97]. PC3 cells stimulated with IGF-1 or phorbol esthers, show PTOV1 and RACK1 colocalized at membrane ruffles [33]. RACK1 interacted with full-length PTOV1 and with the B domain, but not with the A domain, suggesting un-equivalent functions between the two PTOV1 domains. The PTOV1-RACK interaction was localized on 40S ribosomes by polysome profiling experiments in PC3 cells. Significantly, PTOV1 failed to co-sediment with the 40S ribosomal subunit in RACK1 knockdown cells, indicating that RACK1 is necessary for the association of PTOV1 with ribosomes [33]. In addition, PTOV1 co-immunoprecipitated and colocalized with the ribosomal protein S6 (RPS6) corroborating its interaction with ribosomes. Of notice, PTOV1 was not detected in gradient fractions corresponding to polysomes, suggesting that its action is directed to translation initiation rather than elongation. In addition, the overexpression of PTOV1 caused a significant stimulation of bulk protein synthesis, including of c-Jun. In turn, increased levels of PTOV1 and c-Jun induced *SNAI1* transcription, promoted an epithelial-mesenchymal-transition (EMT) and a significant increase of cell invasiveness *in vitro* and tumor growth and metastasis *in vivo* [33]. These findings indicate that the increased expression of PTOV1 in cancer and its ability to bind to RACK1 on ribosomes and to increase protein synthesis is one efficient way for this protein to promote cancer progression.

**Figure 4:**
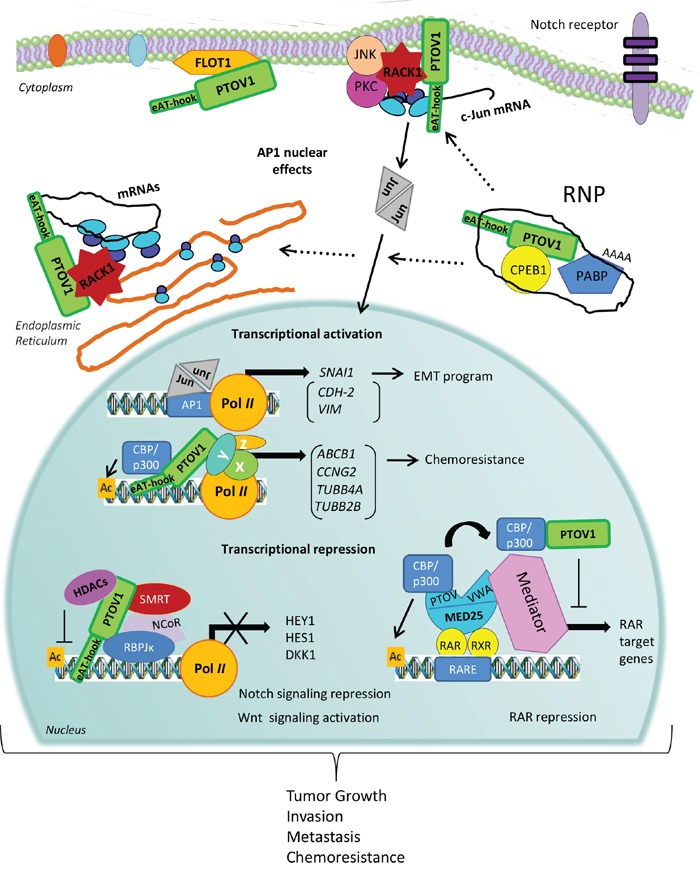
A mechanistic model for PTOV1 actions in cancer progression PTOV1 can shuttle from the cytoplasm to the nucleus during the progression of the cell cycle. In the cytoplasm, PTOV1 is found at perinuclear and submembranes regions associated to Flotillin-1 and also RACK1 and ribosomes. The latter, likely occur in RNA-protein complexes (RNP) that modulate mRNA translation, including the synthesis of the oncogene c-Jun. In the nucleus, PTOV1 can regulate the expression of a number of genes related to cell proliferation, survival, EMT, and chemoresistance, by direct or indirect (genes shown in parenthesis) association to specific promoters to activate or repress transcription. In turn, c-Jun/AP1 may be directly or indirectly contributing to the action of PTOV1 as a transcription factor. PTOV1 is a repressor for the regulation of *HES1, HEY1* and *DKK1*. These effects result in the negative regulation of Notch signaling in PC and the activation of Wnt/β-catenin signaling in breast cancer. In both tumors, these effects culminate with increased tumor growth, invasion, metastasis, and chemoresistance. PTOV1 as a transcriptional repressor requires the presence of HDACs, suggesting that it is an epigenetic regulator. An additional mechanism for transcriptional repression was suggested for the *RAR* promoter, where PTOV1 sequesters the activator CBP from MED25, and results in suppression of transcription of RAR targets. The action of PTOV1 of sequestering activators from MED25 might be associated to inhibition of other MED25 targets. The recent identification of the nucleic acid-binding motif (e)AT-hook, at the N-terminal region of PTOV1 gives support to its role in regulation of gene expression by direct DNA or RNA binding.

The identification of the N-terminal (e)AT-hook motif in PTOV1 and the observed direct interaction of this motif with RNA chains [18], the structural resemblance of PTOV domains with SPOC domains often present in proteins containing RNA binding motifs [16], and the ability of the protein to bind to 40S ribosomes and regulate the rate of mRNA translation, point to a role for PTOV1 both in the nucleus, regulating gene transcription and in the cytoplasm, conceivably as part of ribonucleoprotein (RNP) complexes that may be critical in regulating translation initiation of at least a set of mRNAs, such as *c-Jun* (Figure 4).

### BUZ/Znf-Ubp domains of the histone deacetylase HDAC6

The BUZ (binder of ubiquitin zinc finger) domain, also known as the Znf-UBP (zinc finger-ubiquitin-specific processing protease domain), is present in a subfamily of ubiquitin-specific processing proteases (USPs), the E3 ubiquitin ligase BRCA1-associated protein 2 (BRAP2) and in the histone deacetylase 6 (HDAC6) [98, 99]. The BUZ domain is a sequence-specific protein-binding module that recognizes the free C-termini of proteins. Through its C-terminal sequence (RGMGG), PTOV1 interacts with the BUZ domain of HDAC6 with a low KD value that required the Gly-Gly motif present at the C-terminal of HDAC6 [100]. HDAC6 although it has been detected in the nucleus, it is mostly a cytoplasmic deacetylase that catalyzes the cleavage of the acetyl group of ε-amino groups of lysines and can regulate growth factor-induced chemotaxis by association with the cytoskeleton [101].

The role of HDAC6 in tumor progression is controversial. Some evidences suggest an oncogenic role: its overexpression is associated to mutated K-ras, and correlated with more aggressive tumors and lower survival rate in several tumor types [102–106]. In contrast, several reports suggest a *tumor-suppressor-like* function, where its reduced nuclear localization was associated with distant metastasis, worse overall survival, and poor prognosis [107, 108]. Because PTOV1 is a promoter of motility and invasion, the above observations would be in line with a role for the PTOV1-HDAC6 interaction in the cytoplasm in promoting the pro-oncogenic role of PTOV1. We speculate that, by interacting with HDAC6, PTOV1 might have a more direct access to the cytoskeleton and act in promoting cell motility. Further investigation would be required to ascertain the contribution of HDAC6 to the oncogenic capacities of PTOV1.

### Flotillin-1

PTOV1 was shown to interact with the lipid-raft-associated protein Flotillin-1 [22] a protein that belongs to the Reggie/Flotillin family. Lipid rafts play a central role in membrane trafficking and signaling [109]. Flotillin-1, localized to non-caveolar lipid-rafts [110], has been involved in neuronal regeneration [111], and in insulin signaling in adipocytes, where it generates a signal crucial in the regulation of glucose uptake in adipocytes [112].

In PC cells, Flotillin-1 interacts with the B domain of PTOV1, as expected the two proteins colocalized in lipid rafts and, surprisingly, in the nucleus [22]. After a mitogenic stimulus, Flotillin-1 entered the nucleus concomitantly with PTOV1 shortly before the beginning of the S phase. The overexpression of Flotillin-1 caused a significant increase in cell proliferation, and both PTOV1 and Flotillin-1 are required for PC proliferation. However, while the presence of PTOV1 and an intact carboxy terminus of Flotillin-1 are required for its nuclear entry, the depletion of Flotillin-1 did not affect the nuclear localization of PTOV1 [22]. In additional work, we have shown that Flotillin-1 is required for the stability and function of the Aurora B kinase in mitosis [113]. These data suggest that PTOV1 may drive PC progression in part through the regulation of expression and nuclear localization of Flotillin-1 necessary to support Aurora B kinase mitotic function.

Additional recent reports confirmed the pro-oncogenic effects of increased Flotillin-1 levels in tumors [114–116] and its action in promoting invasion and metastasis through EMT, activation of NF-kB, Wnt/β-catenin and TGF-β pathways [114, 115]. Flotillin-1 is an important regulator of H-ras activation and invasion in triple-negative breast cancer and its expression inversely correlates with patient disease-free survival rates [116]. In gastric cancer, Flotillin-1 overexpression was shown to be the result of miR-485-5p downregulation that correlated with poor prognosis [117]. These findings suggest that the interaction of PTOV1 with Flotillin-1 might amplify their action in tumor progression.

## DISCUSSION: A MODEL FOR PTOV1 ACTIONS

The hitherto little known oncogenic PTOV1 protein has gained recent attention as a regulator of multiple cellular functions and pathways that tend to enhance cell growth and self-renewal in multiple cell types. Here, we propose a mechanistic model that collects the most recently described actions of PTOV1 (Figure 4). PTOV1 shows dual functions in the regulation of gene expression at transcriptional and translational levels. For the role as transcription factor, PTOV1 is found associated with the regulatory regions of at least three genes where it recruits deacetylases and other DNA modifying enzymes to modulate transcription. The association with transcriptional repressor complexes, including several HDACs and NCoR, has been reported for the repression of a few genes (*HES1, HEY1*, and *DKK1*). Transcriptional repression by PTOV1 might also occur through association with activators, such as CBP in the *RARβ2* gene. In this case, the complex PTOV1-CBP is not bound to chromatin, but the protein competes with MED25 for binding and sequestering the activator away from the Polymerase II complex. Conceivably, the association CBP-PTOV1 could also act promoting transcription in different genes.

Additionally, PTOV1 was discovered to regulate protein synthesis by direct association with RACK1 and 40S ribosomes in translation pre-initiation complexes. In PTOV1 overexpressing cells significantly more c-Jun mRNA levels were loaded on polysomes compared to actin, but PTOV1 is not found in polysomal fractions, suggesting that its action is directed to translation initiation, possibly mediating the recruitment of specific mRNA-protein complexes to ribosomes.

Significant for these dual functions described for PTOV1 is the very recent identification of the N-terminal (e)AT-hook motif that, allowing the protein to bind directly to nucleic acids, gives support to the roles in gene expression regulation by direct DNA or RNA binding. Nucleic acids binding through the (e)AT-hook would feasibly allow simultaneous interactions of PTOV1 with different factors binding the A and B domains. For example, protein complexes associated to promoters, or ribonucleoprotein complexes charged with specific mRNAs to be translated. Interestingly, the subcellular localization of the specific interactions mediated by the A domain with HDAC1 and RBP-jκ, are coupled to actions in the regulation of transcription [32]. In contrast, the interactions with cytoplasmic RACK1 and Flotillin-1 exclusively engaging the B domain are linked to activities more closely related to protein synthesis. All together these evidences suggest that PTOV1 might be a new moonlighting protein able to perform different activities in the cell [118], and may utilize separate protein surfaces for its multiple actions [119]. The overexpression of a single protein with multiple actions that converge in activation of proliferation, survival and drug-resistance is energetically convenient for a tumor cell, and might contribute to the acquisition of stemness features. Many questions remain to be solved to understand what is the biological role of PTOV1 in normal tissues, or how does the protein promote tumor progression. The evidences so far suggest that the potential therapeutic effects of its specific targeting in aggressive cancer cells is worthy of being studied.

## References

[1] Benedit P, Paciucci R, Thomson TM, Valeri M, Nadal M, Caceres C, de Torres I, Estivill X, Lozano JJ, Morote J, Reventos J (2001). PTOV1, a novel protein overexpressed in prostate cancer containing a new class of protein homology blocks. Oncogene.

[2] Diamandis EP, Yousef GM, Luo LY, Magklara A, Obiezu CV (2000). The new human kallikrein gene family: implications in carcinogenesis. Trends Endocrinol Metab.

[3] Simoes VL, Alves MG, Martins AD, Dias TR, Rato L, Socorro S, Oliveira PF (2013). Regulation of apoptotic signaling pathways by 5alpha-dihydrotestosterone and 17beta-estradiol in immature rat Sertoli cells. J Steroid Biochem Mol Biol.

[4] Yousef GM, Scorilas A, Diamandis EP (2000). Genomic organization, mapping, tissue expression, and hormonal regulation of trypsin-like serine protease (TLSP PRSS20), a new member of the human kallikrein gene family. Genomics.

[5] Yousef GM, Luo LY, Scherer SW, Sotiropoulou G, Diamandis EP (1999). Molecular characterization of zyme/protease M/neurosin (PRSS9), a hormonally regulated kallikrein-like serine protease. Genomics.

[6] Aken BL, Ayling S, Barrell D, Clarke L, Curwen V, Fairley S, J Fernandez Banet, Billis K, C Garcia Giron, Hourlier T, Howe K, Kahari A, Kokocinski F, Martin FJ, Murphy DN, Nag R (2016). The Ensembl gene annotation system. Database (Oxford).

[7] Casamassimi A, Napoli C (2007). Mediator complexes and eukaryotic transcription regulation: an overview. Biochimie.

[8] Wang C, McCarty IM, Balazs L, Li Y, Steiner MS (2002). A prostate-derived cDNA that is mapped to human chromosome 19 encodes a novel protein. Biochem Biophys Res Commun.

[9] Tomomori-Sato C, Sato S, Parmely TJ, Banks CA, Sorokina I, Florens L, Zybailov B, Washburn MP, Brower CS, Conaway RC, Conaway JW (2004). A mammalian mediator subunit that shares properties with Saccharomyces cerevisiae mediator subunit Cse2. J Biol Chem.

[10] Bourbon HM, Aguilera A, Ansari AZ, Asturias FJ, Berk AJ, Bjorklund S, Blackwell TK, Borggrefe T, Carey M, Carlson M, Conaway JW, Conaway RC, Emmons SW, Fondell JD, Freedman LP, Fukasawa T (2004). A unified nomenclature for protein subunits of mediator complexes linking transcriptional regulators to RNA polymerase II. Mol Cell.

[11] Yang F, DeBeaumont R, Zhou S, Naar AM (2004). The activator-recruited cofactor/Mediator coactivator subunit ARC92 is a functionally important target of the VP16 transcriptional activator. Proc Natl Acad Sci U S A.

[12] Nakamura Y, Suzuki T, Igarashi K, Kanno J, Furukawa T, Tazawa C, Fujishima F, Miura I, Ando T, Moriyama N, Moriya T, Saito H, Yamada S, Sasano H (2006). PTOV1: a novel testosterone-induced atherogenic gene in human aorta. J Pathol.

[13] Schwede T, Kopp J, Guex N, Peitsch MC (2003). SWISS-MODEL: An automated protein homology-modeling server. Nucleic Acids Res.

[14] Bontems F, Verger A, Dewitte F, Lens Z, Baert JL, Ferreira E, de Launoit Y, Sizun C, Guittet E, Villeret V, Monte D (2010). NMR structure of the human Mediator MED25 ACID domain. J Struct Biol.

[15] Eletsky A, Ruyechan WT, Xiao R, Acton TB, Montelione GT, Szyperski T (2011). Solution NMR structure of MED25(391-543) comprising the activator-interacting domain (ACID) of human mediator subunit 25. J Struct Funct Genomics.

[16] Vojnic E, Mourao A, Seizl M, Simon B, Wenzeck L, Lariviere L, Baumli S, Baumgart K, Meisterernst M, Sattler M, Cramer P (2011). Structure and VP16 binding of the Mediator Med25 activator interaction domain. Nat Struct Mol Biol.

[17] Oswald F, Winkler M, Cao Y, Astrahantseff K, Bourteele S, Knochel W, Borggrefe T (2005). RBP-Jkappa/SHARP recruits CtIP/CtBP corepressors to silence Notch target genes. Mol Cell Biol.

[18] Filarsky M, Zillner K, Araya I, Villar-Garea A, Merkl R, Langst G, Nemeth A (2015). The extended AT-hook is a novel RNA binding motif. RNA Biol.

[19] Banks GC, Mohr B, Reeves R (1999). The HMG-I(Y) AT-hook peptide motif confers DNA-binding specificity to a structured chimeric protein. J Biol Chem.

[20] Cairns BR, Schlichter A, Erdjument-Bromage H, Tempst P, Kornberg RD, Winston F (1999). Two functionally distinct forms of the RSC nucleosome-remodeling complex, containing essential AT hook, BAH, and bromodomains. Mol Cell.

[21] Santamaria A, Fernandez PL, Farre X, Benedit P, Reventos J, Morote J, Paciucci R, Thomson TM (2003). PTOV-1, a novel protein overexpressed in prostate cancer, shuttles between the cytoplasm and the nucleus and promotes entry into the S phase of the cell division cycle. Am J Pathol.

[22] Santamaria A, Castellanos E, Gomez V, Benedit P, Renau-Piqueras J, Morote J, Reventos J, Thomson TM, Paciucci R (2005). PTOV1 enables the nuclear translocation and mitogenic activity of flotillin-1, a major protein of lipid rafts. Mol Cell Biol.

[23] Welsh JB, Sapinoso LM, Su AI, Kern SG, Wang-Rodriguez J, Moskaluk CA, Frierson HF, Hampton GM (2001). Analysis of gene expression identifies candidate markers and pharmacological targets in prostate cancer. Cancer Res.

[24] Yao YW, Shi Y, Jia ZF, Jiang YH, Gu Z, Wang J, Aljofan M, Sun ZG (2011). PTOV1 is associated with UCH-L1 and in response to estrogen stimuli during the mouse oocyte development. Histochem Cell Biol.

[25] Guo F, Feng L, Hu JL, Wang ML, Luo P, Zhong XM, Deng AM (2015). Increased PTOV1 expression is related to poor prognosis in epithelial ovarian cancer. Tumour Biol.

[26] Chen S-P, Zhang L-S, Fu B-S, Zeng X-C, Yi H-M, Jiang N (2015). Prostate tumor overexpressed 1 is a novel prognostic marker for hepatocellular carcinoma progression and overall patient survival. Medicine (Baltimore).

[27] Lei F, Zhang L, Li X, Lin X, Wu S, Li F, Liu J (2014). Overexpression of prostate tumor overexpressed 1 correlates with tumor progression and predicts poor prognosis in breast cancer. BMC cancer.

[28] Morote J, Fernandez S, Alana L, Iglesias C, Planas J, Reventos J, Ramon YCS, Paciucci R, de Torres IM (2008). PTOV1 expression predicts prostate cancer in men with isolated high-grade prostatic intraepithelial neoplasia in needle biopsy. Clin Cancer Res.

[29] Scarpelli M, Mazzucchelli R, Barbisan F, Santinelli A, Lopez-Beltran A, Cheng L, Montironi R (2012). Is there a role for prostate tumour overexpressed-1 in the diagnosis of HGPIN and of prostatic adenocarcinoma? A comparison with alpha-methylacyl CoA racemase. Int J Immunopathol Pharmacol.

[30] Mazzucchelli R, Barbisan F, Santinelli A, Lopez-Beltran A, Cheng L, Scarpelli M, Montironi R (2011). Immunohistochemical expression of prostate tumor overexpressed 1 in cystoprostatectomies with incidental and insignificant prostate cancer. Further evidence for field effect in prostatic carcinogenesis. Hum Pathol.

[31] Mazzucchelli R, Scarpelli M, Barbisan F, Santinelli A, Lopez-Beltran A, Cheng L, Montironi R (2013). Immunohistochemical expression of prostate tumour overexpressed 1 (PTOV1) in atypical adenomatous hyperplasia (AAH) of the prostate: additional evidence linking (AAH) to adenocarcinoma. Cell Oncol (Dordr).

[32] Alana L, Sese M, Canovas V, Punyal Y, Fernandez Y, Abasolo I, de Torres I, Ruiz C, Espinosa L, Bigas A, SR Y Cajal, Fernandez PL, Serras F, Corominas M, Thomson TM, Paciucci R (2014). Prostate tumor OVerexpressed-1 (PTOV1) down-regulates HES1 and HEY1 notch targets genes and promotes prostate cancer progression. Mol Cancer.

[33] Marques N, Sese M, Canovas V, Valente F, Bermudo R, de Torres I, Fernandez Y, Abasolo I, Fernandez PL, Contreras H, Castellon E, Celia-Terrassa T, Mendez R, Ramon YCS, Thomson TM, Paciucci R (2014). Regulation of protein translation and c-Jun expression by prostate tumor overexpressed 1. Oncogene.

[34] Cerami E, Gao J, Dogrusoz U, Gross BE, Sumer SO, Aksoy BA, Jacobsen A, Byrne CJ, Heuer ML, Larsson E, Antipin Y, Reva B, Goldberg AP, Sander C, Schultz N (2012). The cBio cancer genomics portal: an open platform for exploring multidimensional cancer genomics data. Cancer Discov.

[35] Gao J, Aksoy BA, Dogrusoz U, Dresdner G, Gross B, Sumer SO, Sun Y, Jacobsen A, Sinha R, Larsson E, Cerami E, Sander C, Schultz N (2013). Integrative analysis of complex cancer genomics and clinical profiles using the cBioPortal. Sci Signal.

[36] Beltran H, Prandi D, Mosquera JM, Benelli M, Puca L, Cyrta J, Marotz C, Giannopoulou E, Chakravarthi BV, Varambally S, Tomlins SA, Nanus DM, Tagawa ST, Van Allen EM, Elemento O, Sboner A (2016). Divergent clonal evolution of castration-resistant neuroendocrine prostate cancer. Nat Med.

[37] Beltran H, Rickman DS, Park K, Chae SS, Sboner A, MacDonald TY, Wang Y, Sheikh KL, Terry S, Tagawa ST, Dhir R, Nelson JB, A de la Taille, Allory Y, Gerstein MB, Perner S (2011). Molecular characterization of neuroendocrine prostate cancer and identification of new drug targets. Cancer Discov.

[38] Grigore AD, Ben-Jacob E, Farach-Carson MC (2015). Prostate cancer and neuroendocrine differentiation: more neuronal, less endocrine?. Front Oncol.

[39] Yang Q, Lin H, Wu S, Lei F, Zhu X, Song L, Hong M, Guo L (2015). Prostate Tumor Overexpressed 1 (PTOV1) Is a Novel Prognostic Marker for Nasopharyngeal Carcinoma Progression and Poor Survival Outcomes. PLoS One.

[40] Fernandez S, Mosquera JL, Alana L, Sanchez-Pla A, Morote J, Ramon YCS, Reventos J, de Torres I, Paciucci R (2011). PTOV1 is overexpressed in human high-grade malignant tumors. Virchows Arch.

[41] Yang L, Wang H, Wang Y, He Z, Chen H, Liang S, He S, Wu S, Song L, Chen Y (2016). Prostate tumor overexpressed-1 in conjunction with human papillomavirus status, predicts outcome in early-stage human laryngeal squamous cell carcinoma. Oncotarget.

[42] Rausch S, Hennenlotter J, Scharpf M, Teepe K, Kuhs U, Aufderklamm S, Bier S, Mischinger J, Gakis G, Stenzl A, Schwentner C, Todenhofer T (2016). Prostate tumor overexpressed 1 expression in invasive urothelial carcinoma. J Cancer Res Clin Oncol.

[43] Liu H, Li J, Zhou Y, Hu Q, Zeng Y, Mohammadreza MM (2016). Human papillomavirus as a favorable prognostic factor in a subset of head and neck squamous cell carcinomas: A meta-analysis. J Med Virol.

[44] Chakravarthy A, Henderson S, Thirdborough SM, Ottensmeier CH, Su X, Lechner M, Feber A, Thomas GJ, Fenton TR (2016). Human Papillomavirus Drives Tumor Development Throughout the Head and Neck: Improved Prognosis Is Associated With an Immune Response Largely Restricted to the Oropharynx. J Clin Oncol.

[45] Wittekindt C, Klussmann JP (2017). Tumor Staging and HPV-Related Oropharyngeal Cancer. Recent Results Cancer Res.

[46] Reva B, Antipin Y, Sander C (2011). Predicting the functional impact of protein mutations: application to cancer genomics. Nucleic Acids Res.

[47] Lee HK, Park UH, Kim EJ, Um SJ (2007). MED25 is distinct from TRAP220/MED1 in cooperating with CBP for retinoid receptor activation. EMBO J.

[48] Verger A, Baert JL, Verreman K, Dewitte F, Ferreira E, Lens Z, de Launoit Y, Villeret V, Monte D (2013). The Mediator complex subunit MED25 is targeted by the N-terminal transactivation domain of the PEA3 group members. Nucleic Acids Res.

[49] Han EH, Rha GB, Chi YI (2012). MED25 is a mediator component of HNF4alpha-driven transcription leading to insulin secretion in pancreatic beta-cells. PLoS One.

[50] Sela D, Conkright JJ, Chen L, Gilmore J, Washburn MP, Florens L, Conaway RC, Conaway JW (2013). Role for human mediator subunit MED25 in recruitment of mediator to promoters by endoplasmic reticulum stress-responsive transcription factor ATF6alpha. J Biol Chem.

[51] Youn HS, Park UH, Kim EJ, Um SJ (2011). PTOV1 antagonizes MED25 in RAR transcriptional activation. Biochem Biophys Res Commun.

[52] Lee B, Wu CY, Lin YW, Park SW, Wei LN (2016). Synergistic activation of Arg1 gene by retinoic acid and IL-4 involves chromatin remodeling for transcription initiation and elongation coupling. Nucleic Acids Res.

[53] Simoni D, Tolomeo M (2001). Retinoids, apoptosis and cancer. Curr Pharm Des.

[54] Shilkaitis A, Green A, Christov K (2015). Retinoids induce cellular senescence in breast cancer cells by RAR-beta dependent and independent pathways: Potential clinical implications (Review). Int J Oncol.

[55] Freemantle SJ, Spinella MJ, Dmitrovsky E (2003). Retinoids in cancer therapy and chemoprevention: promise meets resistance. Oncogene.

[56] Goodman RH, Smolik S (2000). CBP/p300 in cell growth, transformation, and development. Genes Dev.

[57] Youn H, Kim EJ, Um SJ (2013). Zyxin cooperates with PTOV1 to confer retinoic acid resistance by repressing RAR activity. Cancer Lett.

[58] Artavanis-Tsakonas S, Rand MD, Lake RJ (1999). Notch signaling: cell fate control and signal integration in development. Science.

[59] Brou C, Logeat F, Gupta N, Bessia C, LeBail O, Doedens JR, Cumano A, Roux P, Black RA, Israel A (2000). A novel proteolytic cleavage involved in Notch signaling: the role of the disintegrin-metalloprotease TACE. Mol Cell.

[60] Jarriault S, Brou C, Logeat F, Schroeter EH, Kopan R, Israel A (1995). Signalling downstream of activated mammalian Notch. Nature.

[61] Lai EC (2002). Keeping a good pathway down: transcriptional repression of Notch pathway target genes by CSL proteins. EMBO Rep.

[62] Klinakis A, Lobry C, Abdel-Wahab O, Oh P, Haeno H, Buonamici S, van De Walle I, Cathelin S, Trimarchi T, Araldi E, Liu C, Ibrahim S, Beran M, Zavadil J, Efstratiadis A, Taghon T (2011). A novel tumour-suppressor function for the Notch pathway in myeloid leukaemia. Nature.

[63] Lu J, Xia Y, Chen K, Zheng Y, Wang J, Lu W, Yin Q, Wang F, Zhou Y, Guo C (2016). Oncogenic role of the Notch pathway in primary liver cancer. Oncol Lett.

[64] Giachino C, Boulay JL, Ivanek R, Alvarado A, Tostado C, Lugert S, Tchorz J, Coban M, Mariani L, Bettler B, Lathia J, Frank S, Pfister S, Kool M, Taylor V (2015). A Tumor Suppressor Function for Notch Signaling in Forebrain Tumor Subtypes. Cancer Cell.

[65] Wang Z, Li Y, Banerjee S, Kong D, Ahmad A, Nogueira V, Hay N, Sarkar FH (2010). Down-regulation of Notch-1 and Jagged-1 inhibits prostate cancer cell growth, migration and invasion, and induces apoptosis via inactivation of Akt, mTOR, and NF-kappaB signaling pathways. J Cell Biochem.

[66] Whelan JT, Kellogg A, Shewchuk BM, Hewan-Lowe K, Bertrand FE (2009). Notch-1 signaling is lost in prostate adenocarcinoma and promotes PTEN gene expression. J Cell Biochem.

[67] Liu C, Li Z, Bi L, Li K, Zhou B, Xu C, Huang J, Xu K (2014). NOTCH1 signaling promotes chemoresistance via regulating ABCC1 expression in prostate cancer stem cells. Mol Cell Biochem.

[68] Ramos YF, Hestand MS, Verlaan M, Krabbendam E, Ariyurek Y, van Galen M, van Dam H, van Ommen GJ, den Dunnen JT, Zantema A, t Hoen PA (2010). Genome-wide assessment of differential roles for p300 and CBP in transcription regulation. Nucleic Acids Res.

[69] Oswald F, Kostezka U, Astrahantseff K, Bourteele S, Dillinger K, Zechner U, Ludwig L, Wilda M, Hameister H, Knochel W, Liptay S, Schmid RM (2002). SHARP is a novel component of the Notch/RBP-Jkappa signalling pathway. EMBO J.

[70] Kolev V, Mandinova A, Guinea-Viniegra J, Hu B, Lefort K, Lambertini C, Neel V, Dummer R, Wagner EF, Dotto GP (2008). EGFR signalling as a negative regulator of Notch1 gene transcription and function in proliferating keratinocytes and cancer. Nat Cell Biol.

[71] Nicolas M, Wolfer A, Raj K, Kummer JA, Mill P, van Noort M, Hui CC, Clevers H, Dotto GP, Radtke F (2003). Notch1 functions as a tumor suppressor in mouse skin. Nat Genet.

[72] Hu B, Castillo E, Harewood L, Ostano P, Reymond A, Dummer R, Raffoul W, Hoetzenecker W, Hofbauer GF, Dotto GP (2012). Multifocal epithelial tumors and field cancerization from loss of mesenchymal CSL signaling. Cell.

[73] Ai L, Tao Q, Zhong S, Fields CR, Kim WJ, Lee MW, Cui Y, Brown KD, Robertson KD (2006). Inactivation of Wnt inhibitory factor-1 (WIF1) expression by epigenetic silencing is a common event in breast cancer. Carcinogenesis.

[74] Cui Y, Ma W, Lei F, Li Q, Su Y, Lin X, Lin C, Zhang X, Ye L, Wu S, Li J, Yuan Z, Song L (2016). Prostate tumour overexpressed-1 promotes tumourigenicity in human breast cancer via activation of Wnt/beta-catenin signalling. The J Pathol.

[75] Vilchez V, Turcios L, Marti F, Gedaly R (2016). Targeting Wnt/beta-catenin pathway in hepatocellular carcinoma treatment. J Gastroenterol.

[76] Na Y, Lee SM, Kim DS, Park JY (2012). Promoter methylation of Wnt antagonist DKK1 gene and prognostic value in Korean patients with non-small cell lung cancers. Cancer Biomark.

[77] Rawson JB, Manno M, Mrkonjic M, Daftary D, Dicks E, Buchanan DD, Younghusband HB, Parfrey PS, Young JP, Pollett A, Green RC, Gallinger S, McLaughlin JR, Knight JA, Bapat B (2011). Promoter methylation of Wnt antagonists DKK1 and SFRP1 is associated with opposing tumor subtypes in two large populations of colorectal cancer patients. Carcinogenesis.

[78] Suzuki H, Toyota M, Carraway H, Gabrielson E, Ohmura T, Fujikane T, Nishikawa N, Sogabe Y, Nojima M, Sonoda T, Mori M, Hirata K, Imai K, Shinomura Y, Baylin SB, Tokino T (2008). Frequent epigenetic inactivation of Wnt antagonist genes in breast cancer. Br J Cancer.

[79] Wang ZA, Shen MM (2011). Revisiting the concept of cancer stem cells in prostate cancer. Oncogene.

[80] Feinberg AP, Ohlsson R, Henikoff S (2006). The epigenetic progenitor origin of human cancer. Nat Rev Genet.

[81] Hurt EM, Kawasaki BT, Klarmann GJ, Thomas SB, Farrar WL (2008). CD44+ CD24(−) prostate cells are early cancer progenitor/stem cells that provide a model for patients with poor prognosis. Br J Cancer.

[82] Duhagon MA, Hurt EM, Sotelo-Silveira JR, Zhang X, Farrar WL (2010). Genomic profiling of tumor initiating prostatospheres. BMC genomics.

[83] Chandrasekar T, Yang JC, Gao AC, Evans CP (2015). Mechanisms of resistance in castration-resistant prostate cancer (CRPC). Trans Androl Urol.

[84] Ron D, Chen CH, Caldwell J, Jamieson L, Orr E, Mochly-Rosen D (1994). Cloning of an intracellular receptor for protein kinase C: a homolog of the beta subunit of G proteins. Proc Natl Acad Sci U S A.

[85] Mochly-Rosen D, Smith BL, Chen CH, Disatnik MH, Ron D (1995). Interaction of protein kinase C with RACK1, a receptor for activated C-kinase: a role in beta protein kinase C mediated signal transduction. Biochem Soc Trans.

[86] Arimoto K, Fukuda H, Imajoh-Ohmi S, Saito H, Takekawa M (2008). Formation of stress granules inhibits apoptosis by suppressing stress-responsive MAPK pathways. Nat Cell Biol.

[87] Kiely PA, Sant A, O'Connor R (2002). RACK1 is an insulin-like growth factor 1 (IGF-1) receptor-interacting protein that can regulate IGF-1-mediated Akt activation and protection from cell death. J Biol Chem.

[88] Zhang W, Zong CS, Hermanto U, Lopez-Bergami P, Ronai Z, Wang LH (2006). RACK1 recruits STAT3 specifically to insulin and insulin-like growth factor 1 receptors for activation, which is important for regulating anchorage-independent growth. Mol Cell Biol.

[89] Sengupta J, Nilsson J, Gursky R, Spahn CM, Nissen P, Frank J (2004). Identification of the versatile scaffold protein RACK1 on the eukaryotic ribosome by cryo-EM. Nat Struct Mol Biol.

[90] Sharma G, Pallesen J, Das S, Grassucci R, Langlois R, Hampton CM, Kelly DF, des Georges A, Frank J (2013). Affinity grid-based cryo-EM of PKC binding to RACK1 on the ribosome. J Struct Biol.

[91] Gandin V, Miluzio A, Barbieri AM, Beugnet A, Kiyokawa H, Marchisio PC, Biffo S (2008). Eukaryotic initiation factor 6 is rate-limiting in translation, growth and transformation. Nature.

[92] Lopez-Bergami P, Habelhah H, Bhoumik A, Zhang W, Wang LH, Ronai Z (2005). RACK1 mediates activation of JNK by protein kinase C [corrected]. Mol Cell.

[93] Gandin V, Gutierrez GJ, Brill LM, Varsano T, Feng Y, Aza-Blanc P, Au Q, McLaughlan S, Ferreira TA, Alain T, Sonenberg N, Topisirovic I, Ronai ZA (2013). Degradation of newly synthesized polypeptides by ribosome-associated RACK1/c-Jun N-terminal kinase/eukaryotic elongation factor 1A2 complex. Mol Cell Biol.

[94] Berns H, Humar R, Hengerer B, Kiefer FN, Battegay EJ (2000). RACK1 is up-regulated in angiogenesis and human carcinomas. FASEB J.

[95] Cao XX, Xu JD, Liu XL, Xu JW, Wang WJ, Li QQ, Chen Q, Xu ZD, Liu XP (2010). RACK1: A superior independent predictor for poor clinical outcome in breast cancer. Int J Cancer.

[96] Nagashio R, Sato Y, Matsumoto T, Kageyama T, Satoh Y, Shinichiro R, Masuda N, Goshima N, Jiang SX, Okayasu I (2010). Expression of RACK1 is a novel biomarker in pulmonary adenocarcinomas. Lung Cancer.

[97] Ruan Y, Sun L, Hao Y, Wang L, Xu J, Zhang W, Xie J, Guo L, Zhou L, Yun X, Zhu H, Shen A, Gu J (2012). Ribosomal RACK1 promotes chemoresistance and growth in human hepatocellular carcinoma. J Clin Invest.

[98] Bonnet J, Romier C, Tora L, Devys D (2008). Zinc-finger UBPs: regulators of deubiquitylation. Trends Biochem Sci.

[99] Pai MT, Tzeng SR, Kovacs JJ, Keaton MA, Li SS, Yao TP, Zhou P (2007). Solution structure of the Ubp-M BUZ domain, a highly specific protein module that recognizes the C-terminal tail of free ubiquitin. J Mol Biol.

[100] Hard RL, Liu J, Shen J, Zhou P, Pei D HDAC6 and Ubp-M BUZ domains recognize specific C-terminal sequences of proteins. Biochemistry.

[101] Hubbert C, Guardiola A, Shao R, Kawaguchi Y, Ito A, Nixon A, Yoshida M, Wang XF, Yao TP (2002). HDAC6 is a microtubule-associated deacetylase. Nature.

[102] Kanno K, Kanno S, Nitta H, Uesugi N, Sugai T, Masuda T, Wakabayashi G, Maesawa C (2012). Overexpression of histone deacetylase 6 contributes to accelerated migration and invasion activity of hepatocellular carcinoma cells. Oncology Rep.

[103] Bazzaro M, Lin Z, Santillan A, Lee MK, Wang MC, Chan KC, Bristow RE, Mazitschek R, Bradner J, Roden RB (2008). Ubiquitin proteasome system stress underlies synergistic killing of ovarian cancer cells by bortezomib and a novel HDAC6 inhibitor. Clin Cancer Res.

[104] Bradbury CA, Khanim FL, Hayden R, Bunce CM, White DA, Drayson MT, Craddock C, Turner BM (2005). Histone deacetylases in acute myeloid leukaemia show a distinctive pattern of expression that changes selectively in response to deacetylase inhibitors. Leukemia.

[105] Wang Q, Tan R, Zhu X, Zhang Y, Tan Z, Su B, Li Y (2016). Oncogenic K-ras confers SAHA resistance by up-regulating HDAC6 and c-myc expression. Oncotarget.

[106] Woan KV, Lienlaf M, Perez-Villaroel P, Lee C, Cheng F, Knox T, Woods DM, Barrios K, Powers J, Sahakian E, Wang HW, Canales J, Marante D, Smalley KS, Bergman J, Seto E (2015). Targeting histone deacetylase 6 mediates a dual anti-melanoma effect: Enhanced antitumor immunity and impaired cell proliferation. Mol Oncol.

[107] Yang CJ, Liu YP, Dai HY, Shiue YL, Tsai CJ, Huang MS, Yeh YT (2015). Nuclear HDAC6 inhibits invasion by suppressing NF-kappaB/MMP2 and is inversely correlated with metastasis of non-small cell lung cancer. Oncotarget.

[108] Jung KH, Noh JH, Kim JK, Eun JW, Bae HJ, Chang YG, Kim MG, Park WS, Lee JY, Lee SY, Chu IS, Nam SW (2012). Histone deacetylase 6 functions as a tumor suppressor by activating c-Jun NH2-terminal kinase-mediated beclin 1-dependent autophagic cell death in liver cancer. Hepatology.

[109] Simons K, Gerl MJ (2010). Revitalizing membrane rafts: new tools and insights. Nat Rev Mol Cell Biol.

[110] Lang DM, Lommel S, Jung M, Ankerhold R, Petrausch B, Laessing U, Wiechers MF, Plattner H, Stuermer CA (1998). Identification of reggie-1 and reggie-2 as plasmamembrane-associated proteins which cocluster with activated GPI-anchored cell adhesion molecules in non-caveolar micropatches in neurons. J Neurobiol.

[111] Munderloh C, Solis GP, Bodrikov V, Jaeger FA, Wiechers M, Malaga-Trillo E, Stuermer CA (2009). Reggies/flotillins regulate retinal axon regeneration in the zebrafish optic nerve and differentiation of hippocampal and N2a neurons. J Neurosci.

[112] Baumann CA, Ribon V, Kanzaki M, Thurmond DC, Mora S, Shigematsu S, Bickel PE, Pessin JE, Saltiel AR (2000). CAP defines a second signalling pathway required for insulin-stimulated glucose transport. Nature.

[113] Gomez V, Sese M, Santamaria A, Martinez JD, Castellanos E, Soler M, Thomson TM, Paciucci R (2010). Regulation of aurora B kinase by the lipid raft protein flotillin-1. J Biol Chem.

[114] Cao S, Cui Y, Xiao H, Mai M, Wang C, Xie S, Yang J, Wu S, Li J, Song L, Guo X, Lin C (2016). Upregulation of flotillin-1 promotes invasion and metastasis by activating TGF-beta signaling in nasopharyngeal carcinoma. Oncotarget.

[115] Li Z, Yang Y, Gao Y, Wu X, Yang X, Zhu Y, Yang H, Wu L, Yang C, Song L (2016). Elevated expression of flotillin-1 is associated with lymph node metastasis and poor prognosis in early-stage cervical cancer. Am J Cancer Res.

[116] Koh M, Yong HY, Kim ES, Son H, Jeon YR, Hwang JS, Kim MO, Cha Y, Choi WS, Noh DY, Lee KM, Kim KB, Lee JS, Kim HJ, Kim H, Kim HH (2016). A novel role for flotillin-1 in H-Ras-regulated breast cancer aggressiveness. Int J Cancer.

[117] Kang M, Ren MP, Zhao L, Li CP, Deng MM (2015). miR-485-5p acts as a negative regulator in gastric cancer progression by targeting flotillin-1. Am J Transl Res.

[118] Jeffery CJ (2014). An introduction to protein moonlighting. Biochemical Society transactions.

[119] Jeffery CJ (2004). Molecular mechanisms for multitasking: recent crystal structures of moonlighting proteins. Curr Opin Struct Biol.

[120] Jensen LJ, Kuhn M, Stark M, Chaffron S, Creevey C, Muller J, Doerks T, Julien P, Roth A, Simonovic M, Bork P, von Mering C (2009). STRING 8--a global view on proteins and their functional interactions in 630 organisms. Nucleic Acids Res.

[121] Stark C, Breitkreutz BJ, Reguly T, Boucher L, Breitkreutz A, Tyers M (2006). BioGRID: a general repository for interaction datasets. Nucleic Acids Res.

[122] Behrends C, Sowa ME, Gygi SP, Harper JW (2010). Network organization of the human autophagy system. Nature.

[123] Lu WH, Shi YX, Ma ZL, Wang G, Liu L, Chuai M, Song X, Munsterberg A, Cao L, Yang X (2016). Proper autophagy is indispensable for angiogenesis during chick embryo development. Cell Cycle.

[124] Di Fazio P, Waldegger P, Jabari S, Lingelbach S, Montalbano R, Ocker M, Slater EP, Bartsch DK, Illig R, Neureiter D, Wissniowski TT (2016). Autophagy-related cell death by pan-histone deacetylase inhibition in liver cancer. Oncotarget.

[125] Dyson HJ, Wright PE (2016). Role of Intrinsic Protein Disorder in the Function and Interactions of the Transcriptional Coactivators CREB-binding Protein (CBP) and p300. J Biol Chem.

[126] Sun XJ, Man N, Tan Y, Nimer SD, Wang L (2015). The Role of Histone Acetyltransferases in Normal and Malignant Hematopoiesis. Frontiers in Oncology.

[127] Bennett EJ, Rush J, Gygi SP, Harper JW (2010). Dynamics of cullin-RING ubiquitin ligase network revealed by systematic quantitative proteomics. Cell.

[128] Chen P, Yao GD (2016). The role of cullin proteins in gastric cancer. Tumor Biol.

[129] Stelzl U, Worm U, Lalowski M, Haenig C, Brembeck FH, Goehler H, Stroedicke M, Zenkner M, Schoenherr A, Koeppen S, Timm J, Mintzlaff S, Abraham C, Bock N, Kietzmann S, Goedde A (2005). A human protein-protein interaction network: a resource for annotating the proteome. Cell.

[130] Brandt GS, Bailey S (2013). Dematin, a human erythrocyte cytoskeletal protein, is a substrate for a recombinant FIKK kinase from Plasmodium falciparum. Mol Biochem Parasitol.

[131] Lutchman M, Pack S, Kim AC, Azim A, Emmert-Buck M, van Huffel C, Zhuang Z, Chishti AH (1999). Loss of heterozygosity on 8p in prostate cancer implicates a role for dematin in tumor progression. Cancer Genet Cytogenet.

[132] Ito A, Kawaguchi Y, Lai CH, Kovacs JJ, Higashimoto Y, Appella E, Yao TP (2002). MDM2-HDAC1-mediated deacetylation of p53 is required for its degradation. EMBO J.

[133] Colarossi L, Memeo L, Colarossi C, Aiello E, Iuppa A, Espina V, Liotta L, Mueller C (2014). Inhibition of histone deacetylase 4 increases cytotoxicity of docetaxel in gastric cancer cells. Proteomics Clin Appl.

[134] Wang AH, Bertos NR, Vezmar M, Pelletier N, Crosato M, Heng HH, Th'ng J, Han J, Yang XJ (1999). HDAC4, a human histone deacetylase related to yeast HDA1, is a transcriptional corepressor. Mol Cell Biol.

[135] Seidel C, Schnekenburger M, Dicato M, Diederich M (2015). Histone deacetylase 6 in health and disease. Epigenomics.

[136] Huttlin EL, Ting L, Bruckner RJ, Gebreab F, Gygi MP, Szpyt J, Tam S, Zarraga G, Colby G, Baltier K, Dong R, Guarani V, Vaites LP, Ordureau A, Rad R, Erickson BK (2015). The BioPlex Network: A Systematic Exploration of the Human Interactome. Cell.

[137] Chin KT, Xu HT, Ching YP, Jin DY (2007). Differential subcellular localization and activity of kelch repeat proteins KLHDC1 and KLHDC2. Mol Cell Biochem.

[138] Wong MM, Guo C, Zhang J (2014). Nuclear receptor corepressor complexes in cancer: mechanism, function and regulation. Am J Clin Exp Urol.

[139] Giannopoulou EA, Emmanouilidis L, Sattler M, Dodt G, Wilmanns M (2016). Towards the molecular mechanism of the integration of peroxisomal membrane proteins. Biochim Biophys Acta.

[140] Eichner T, Kutter S, Labeikovsky W, Buosi V, Kern D (2016). Molecular Mechanism of Pin1-Tau Recognition and Catalysis. J Mol Biol.

[141] D'Artista L, Bisso A, Piontini A, Doni M, Verrecchia A, Kress TR, Morelli MJ, G Del Sal, Amati B, Campaner S (2016). Pin1 is required for sustained B cell proliferation upon oncogenic activation of Myc. Oncotarget.

[142] Kim G, Kim JY, Choi HS (2015). Peptidyl-Prolyl cis/trans Isomerase NIMA-Interacting 1 as a Therapeutic Target in Hepatocellular Carcinoma. Biol Pharm Bull.

[143] Hauri S, Wepf A, van Drogen A, Varjosalo M, Tapon N, Aebersold R, Gstaiger M (2013). Interaction proteome of human Hippo signaling: modular control of the co-activator YAP1. Mol Syst Biol.

[144] Iwasa H, Jiang X, Hata Y (2015). the Putative Tumor Suppressor of the RASSF Family. Cancers.

[145] Blanpain C, Lowry WE, Pasolli HA, Fuchs E (2006). Canonical notch signaling functions as a commitment switch in the epidermal lineage. Genes Dev.

[146] Meyuhas O (2015). Ribosomal Protein S6 Phosphorylation: Four Decades of Research. Int Rev Cell Mol Biol.

[147] Chen B, Tan Z, Gao J, Wu W, Liu L, Jin W, Cao Y, Zhao S, Zhang W, Qiu Z, Liu D, Mo X, Li W (2015). Hyperphosphorylation of ribosomal protein S6 predicts unfavorable clinical survival in non-small cell lung cancer. J Exp Clin Cancer Res.

[148] Knoll M, Macher-Goeppinger S, Kopitz J, Duensing S, Pahernik S, Hohenfellner M, Schirmacher P, Roth W (2016). The ribosomal protein S6 in renal cell carcinoma: functional relevance and potential as biomarker. Oncotarget.

[149] Benzinger A, Muster N, Koch HB, Yates JR, Hermeking H (2005). Targeted proteomic analysis of 14-3-3 sigma, a p53 effector commonly silenced in cancer. Mol Cell Proteomics.

[150] Hermeking H, Lengauer C, Polyak K, TC He, Zhang L, Thiagalingam S, Kinzler KW, Vogelstein B (1997). 14-3-3sigma is a p53-regulated inhibitor of G2/M progression. Mol Cell.

[151] Kim S, Wong P, Coulombe PA (2006). A keratin cytoskeletal protein regulates protein synthesis and epithelial cell growth. Nature.

[152] Hinrichsen I, Ernst BP, Nuber F, Passmann S, Schafer D, Steinke V, Friedrichs N, Plotz G, Zeuzem S, Brieger A (2014). Reduced migration of MLH1 deficient colon cancer cells depends on SPTAN1. Mol Cancer.

[153] Tohyama J, Nakashima M, Nabatame S, Gaik-Siew C, Miyata R, Rener-Primec Z, Kato M, Matsumoto N, Saitsu H (2015). SPTAN1 encephalopathy: distinct phenotypes and genotypes. J Hum Genet.

[154] Nathan JA, Kim HT, Ting L, Gygi SP, Goldberg AL (2013). Why do cellular proteins linked to K63-polyubiquitin chains not associate with proteasomes?. EMBO J.

[155] Crinelli R, Bianchi M, Radici L, Carloni E, Giacomini E, Magnani M (2015). Molecular Dissection of the Human Ubiquitin C Promoter Reveals Heat Shock Element Architectures with Activating and Repressive Functions. PLoS One.

[156] Zhuo X, Guo X, Zhang X, Jing G, Wang Y, Chen Q, Jiang Q, Liu J, Zhang C (2015). Usp16 regulates kinetochore localization of Plk1 to promote proper chromosome alignment in mitosis. J Cell Biol.

[157] Hein MY, Hubner NC, Poser I, Cox J, Nagaraj N, Toyoda Y, Gak IA, Weisswange I, Mansfeld J, Buchholz F, Hyman AA, Mann M (2015). A human interactome in three quantitative dimensions organized by stoichiometries and abundances. Cell.

[158] Park GY, Han JY, Han YK, Kim SD, Kim JS, Jo WS, Chun SH, Jeong DH, Lee CW, Yang K, Lee CG (2014). 14-3-3 eta depletion sensitizes glioblastoma cells to irradiation due to enhanced mitotic cell death. Cancer Gene Ther.

[159] Lee CG, Park GY, Han YK, Lee JH, Chun SH, Park HY, Lim KH, Kim EG, Choi YJ, Yang K, Lee CW (2013). Roles of 14-3-3eta in mitotic progression and its potential use as a therapeutic target for cancers. Oncogene.

